# A Comparison of Different Strength Measurement in Taekwondo: Herman Trainer, Manual Tester, and Standing Long Jump

**DOI:** 10.3390/medicina60040550

**Published:** 2024-03-28

**Authors:** Ayşe Hazal Boyanmış, İnci Kesilmiş, Manolya Akın, Buse Yilmaz, Aşina Uslular, Yesim Karac Ocal, Hulya Andre

**Affiliations:** 1 Faculty of Sports Sciences, Mersin University, Mersin 33110, Turkey; incikesilmis@mersin.edu.tr (İ.K.); manolya@mersin.edu.tr (M.A.); yilmazbusee.00@gmail.com (B.Y.); 2 Faculty of Sports Sciences, Kütahya Dumlupınar University, Kütahya 43100, Turkey; a.uslular@gmail.com; 3 Faculty of Sports Sciences, Yozgat Bozok University, Yozgat 66200, Turkey; ysm74@hotmail.com (Y.K.O.); hulya.andre@gmail.com (H.A.)

**Keywords:** taekwondo, Herman trainer, Lafayette manual, standing long jump, strength

## Abstract

*Background and Objective*: The accurate prediction of strength development relies on identifying the most appropriate measurement methods. This study compared diverse strength measurement techniques to assess their effectiveness in predicting strength development. Participants were taekwondo athletes competing at the red–black belt level or above. *Methods*: Technical striking forces (palding, dollyeo chagi, dwit chagi, and yeop chagi) were measured using a Herman Digital Trainer fixed to a striking stand. Quadriceps and hamstring strength were assessed with a Lafayette force measuring device. Explosive leg strength was evaluated through a standing long jump test, normalized for leg length. The Pearson correlation coefficient was used to examine relationships between measurement methods. *Results*: The standing long jump test showed no significant correlation with other strength assessments. A moderate positive correlation was found between Herman digital trainer measurements and Lafayette digital hand-held dynamometer results. A high positive correlation (r = 0.736, *p* < 0.001) emerged between hamstring strength and palding chagi technical strike force results. Technical strike kicks showed a significant positive correlation with each other and, also, a right foot–left foot correlation was observed. *Conclusions*: It was concluded that the standing long jump test, which was shown as one of the explosive leg strength measurement methods in field studies as an alternative to laboratory tests, did not correlate with other strength tests; therefore, this test is weak and insufficient to predict strength skills in taekwondo. In addition, this study showed that the hamstring muscle was more predictive in the measurement of technical strength. In future studies, it might be more useful to measure hamstring muscle strength or technical kick strength instead of a standing long jump field test.

## 1. Introduction

Taekwondo is a martial art that demands exceptional lower extremity muscle strength for optimal kicking, jumping, postural stability, and overall performance [1,2,3]. Strength is usually defined as the peak force or torque developed during a maximal voluntary contraction [4]. Strength in taekwondo translates to powerful strikes, resistance against opponents, and tactical superiority [5]. Due to the sport’s technical structure and competition rules, quick bursts of strength are essential; athletes must deliver intense force with each strike while maintaining consistent power output. The quadriceps and hamstring muscles play a crucial role in lower extremity function, providing joint stability as the knee moves [6]. The quadriceps, in particular, are vital for balance, knee stability, and executing functional movements like kicking [7]. Taekwondo competitors exhibit moderate to high maximum dynamic strength characteristics in the lower and upper extremities. The physiological demands of taekwondo competitions require athletes to be proficient in various aspects of conditioning, including muscular strength and muscular power. Taekwondo athletes need these parameters to effectively perform and sustain technical and tactical actions in a match [8,9,10,11]. Therefore, different strength-building exercises are needed in taekwondo. In recent years, supplementing traditional taekwondo training with additional training methods has improved training efficiency and variety [12,13,14,15,16,17]. While there is no single best method empirically proven to improve striking strength in combat sports, success in taekwondo depends on an athlete’s ability to land more powerful strikes than they receive. Evidence suggests that striking strength can be increased through a combination of both specific and non-specific strength and conditioning techniques [18]. Furthermore, Sousa et al. highlight the importance of strength for technical striking in taekwondo. Their meta-analysis emphasizes improving technical and methodological aspects of training to enhance competition performance [19].

Accurate measurement of performance indicators, like hitting power, is essential for talent identification, training program design, and match result predictions [20,21]. Quantifying performance changes with numerical data enables sports scientists and coaches to differentiate between athletes and track individual progress in response to training [18]. In taekwondo, the evaluation of strength, which is one of the motoric characteristics that can provide an advantage to the athlete in the match, can be done by various methods. Strength plays a crucial role in sporting success and can be assessed using direct and indirect measurement methods. While direct measurement devices like Cybex and Biodex offer precise quantifications of strength gains, their high cost and laboratory-specific nature can be limiting. As an alternative to them, indirect methods such as the abalakov, high jump, and standing long jump are used, which measure explosive leg strength in the field [22,23,24,25]. Even in longitudinal studies such as the Amsterdam Growth study, the standing high jump has been used as an alternative to laboratory tests [26]. Previous research indicates that field tests, such as the standing long jump and vertical jump included in the EUROFIT assessment, offer reliable measures of physical fitness for both research and practical applications [26,27,28]. However, it seems more important to focus on striking force when measuring strength in combat athletes such as taekwondo [18,20,21,29].

Experienced combat athletes demonstrate superior striking force compared to less experienced individuals [30]. However, the impact of established force measurement methods on assessing these forces in sports like taekwondo needs further clarification [29]. A review of striking force measurement in full-contact combat sport reveals no clear “gold standard”; therefore, selecting a strike force monitoring method should prioritize economic and organizational constraints [20,21,29]. To examine lower extremity strength and bilateral/ipsilateral asymmetry in taekwondo athletes, the Lafayette manual muscle tester is a common tool for measuring maximum isometric strength [30]. In martial arts like taekwondo, the magnitude of force applied during technical strikes is crucial. The Herman digital trainer provides valuable data regarding impact velocity, vectorial force distribution, and the acceleration produced during contact with the target (quantified as G-force). This device also tracks and analyzes a series of strikes, offering metrics such as the duration, peak force, total force, average force, and the total number of strikes—all presented graphically and numerically [31]. There is a wide variety of tests to evaluate strength, but not all are relevant to the assessment of strength for sport. The selection must take into account the specific quality to be measured [32].

As a result of hitting the opponent above a certain level of strength, sensors in the protective equipment detect the force and give points to the athlete [33,34]. Continuous monitoring of strength gains is essential for evaluating athlete progress in combat sports, including taekwondo. It is important to determine whether the results of strength measurement methods [35] that coaches can easily apply during training without the need for a laboratory environment show correlations with each other and to understand whether they can be used as an alternative to each other. Identifying the correlation between methods for predicting strength development is, therefore, of paramount importance. 

Coaches invest significant training time in improving athletes’ striking strength. Understanding the effectiveness of different force measurement methods is crucial to assess training progress. However, the scientific community lacks a universally accepted “gold standard” kinetic variable for evaluating hitting performance. While the optimal force variables remain unclear, maximal force measurement is a prevalent approach in research [21]. This study aims to contribute by analyzing results from three distinct measurement tools, the Herman digital trainer, the Lafayette manual tester, and the standing long jump, and exploring the correlations between these devices in active taekwondo athletes. The findings of this research will provide valuable insights for the strength measurement results correlation to each other, ultimately contributing to the body of sports science literature.

## 2. Materials and Methods

### 2.1. Procedure

This study employs an observational research design with a correlational approach, to investigate relationships among three strength measurement methods. Correlational research allows for insightful predictions and seeks to understand naturally occurring relationships between variables without experimental manipulation [36].

This study adhered to strict ethical guidelines. Prior to participation, we obtained a written ethics approval from the Mersin University Ethics Committee (2017/03). All athletes provided a written informed consent in accordance with the Helsinki criteria, having been fully briefed on the study’s purpose, materials, and methodology. The sample of the research was determined by the “convenience sampling” method. Three measurement methods were employed, as follows:

- Herman trainer: Attached to a striking stand, this device specifically measures the force of various taekwondo striking techniques.
- Lafayette manual muscle tester: This portable tool directly assesses quadriceps and hamstring strength.
- Standing long jump: This field test provides a measure of explosive leg strength.

### 2.2. Participants

This study included 31 taekwondo athletes (12 female and 19 male) competing at the red–black belt level or above. Participation was entirely voluntary. Data was collected in April 2018. The target population consisted of all red belt and above taekwondo athletes in Mersin, with the sample group drawn from licensed athletes at Toros and Zirve Sports Clubs. To be included in the study, athletes had to hold a red belt or above in taekwondo and have no recent injury history. Athletes with less than one year of training experience were excluded.

### 2.3. The Herman Digital Trainer Measurements

**Figure 1:**
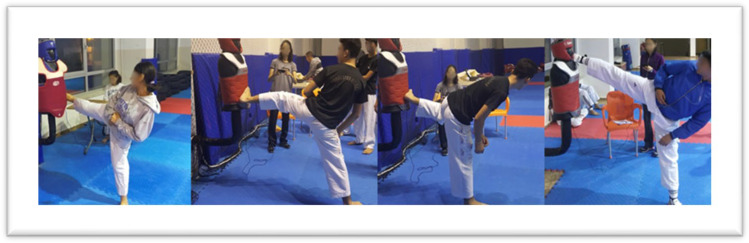
Herman trainer measurements.

**Figure 2:**
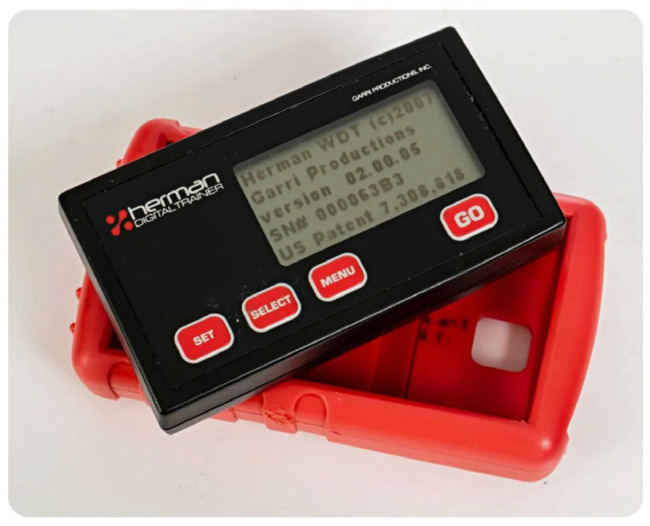
Herman digital trainer device.

The Herman digital trainer measures and records the impact of a preselected number of hits. To measure the intensity of taekwondo technical strokes, we utilized This device, which was securely mounted on a kick stand and consisted of a sensor-equipped box and a display unit. As a warm-up exercise, running and then stretching exercises were first performed, before the structure was completed with classic taekwondo drills. Following a 20 min warm-up (jogging, rope skipping, hip circles, arm circles, neck turns, forward and side bending, knee pulls, knee turns, and stretching), the researcher introduced the device and provided instructions. For adaptation, participants performed two practice trials. Athletes then executed three repetitions of the palding, dollyeo chagi, yeop chagi, and dwit chagi techniques with both their right and left feet [33]. The researcher recorded the three values obtained for each technique, selecting the highest value for analysis [32]. The Herman digital trainer calculates a unit value reflecting the impact force velocity and the vectorial distribution of each strike (Figure 1 and Figure 2). The literature includes examples of the Herman digital trainer being used for striking force measurements; however, its validity and reliability in this context have not been formally investigated [29,33,37].

### 2.4. Lafayette Digital Hand Dynamometer Measurements

The Lafayette hand-held dynamometer is a proven tool for quantifying muscle strength, measuring various force-related metrics including peak force, time to reach peak force, total test time, average force, real-time force, end force, average force from peak to end, and percentage decrease from peak to end force [38,39]. Its versatility and reliability make it a popular choice for force measurement in athletes [40,41]. It has been stated that hand dynameters, such as the Lafayette manual muscle testing device, have become useful in athletic field tests because they are a convenient, portable, non-invasive, relatively fast, easy, and inexpensive way to objectively measure applied forces [38]. The dynamometer has technical limitations due to its design. Since it cannot be fixed during the measurement, there is a possibility that the opposing force applied by the person applying the measurement against the athlete may affect the results. For this reason, there are studies in the literature reporting that it may be a more reliable method in test–retest applications and that it is more appropriate to use it this way [38,39]. Quadriceps and hamstring muscle strength were assessed via a portable Lafayette digital hand dynamometer. This device features interchangeable heads, an liquid crystal display (LCD), and provides data including peak power, time to reach peak power, total test duration, and average force (in kg, Newtons, and pounds). The “make test” isometric contraction protocol was employed, requiring the athlete to exert maximum force against the dynamometer while the researcher held it steadily.

For quadriceps measurements, participants were seated with hips and knees flexed at 90°, and their feet unsupported. The dynamometer was positioned perpendicular to the leg, 1–2 cm above the malleolus. Athletes performed two 5 s maximum voluntary contractions for each leg, with a 1 min rest interval.

For hamstring measurements, participants lay prone with their knees flexed at 90°. The dynamometer was placed perpendicularly 2–3 cm above the malleolus. 

**Figure 3:**
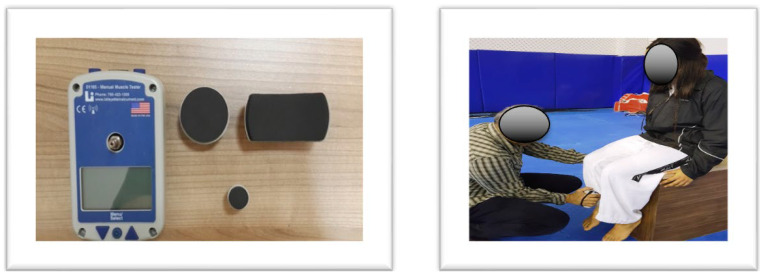
Lafayette digital hand dynamometer and measurement.

For both muscle groups, measurements were taken twice on each leg by the same researcher, with the highest value in librae (lbs)) used for analysis (Figure 3).

### 2.5. Standing Long Jump

**Figure 4:**
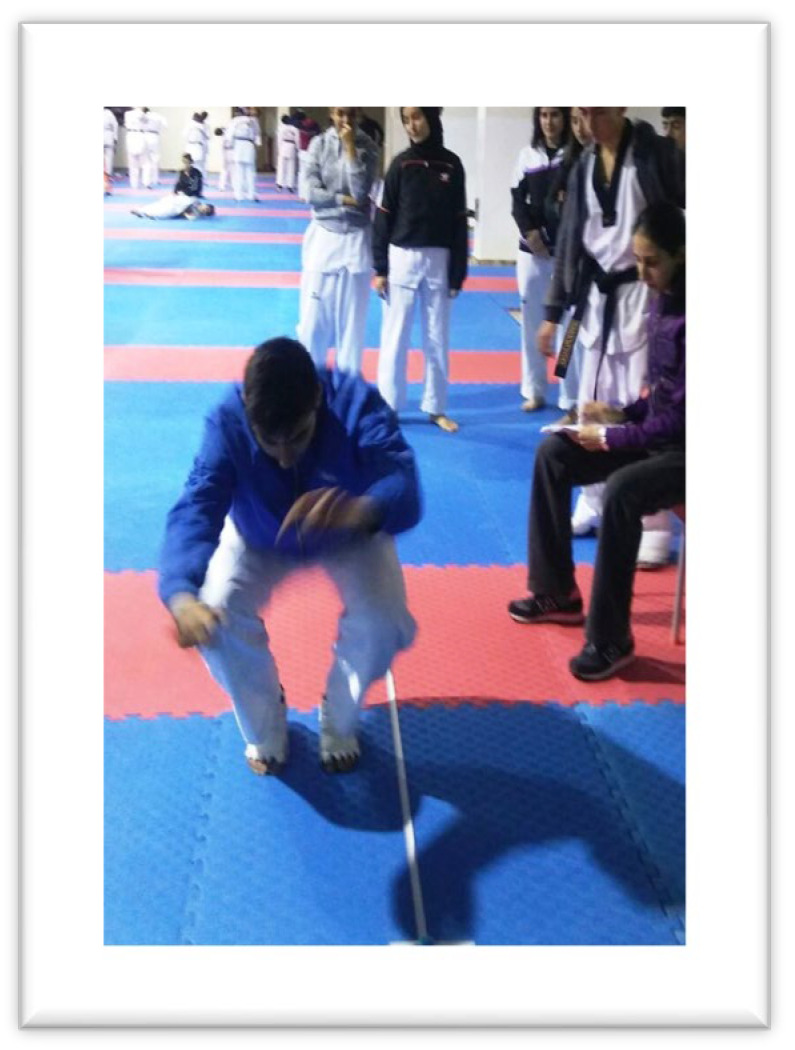
Standing long jump measurement.

The standing long jump is considered a fundamental motor skill for a variety of sports where high-velocity contractions are demanded: sprinting, hurdling, and jumping in athletics, and some combat sports. It is also often used as one of the best functional tests to assess the explosive power of the lower extremity [42]. The standing long jump also reflects the level of human muscle power, which is related to the number of fast-twitch fibers and the cross-sectional area [43]. The standing long jump is divided into four stages, which are pre-swing, take-off, airborne, and landing [44]. Measurement is easy and takes a short time. Athletes were ready at the starting point of a meter glued with tape to the floor, their legs were shoulder-width apart, and their knees were slightly flexed. The athlete jumped forward on the command, and the distance from the heel of the foot to the starting point was recorded in cm. To assess explosive leg strength, a performance ratio was calculated by dividing the jump distance by the athlete’s leg length and multiplying by 100 [45]. Participants were instructed to jump as far as they could (Figure 4).

Adjusted leg length = (Jumping distance/Leg length) × 100

### 2.6. Statistical Analysis

Following descriptive statistics calculations, we assessed the data distribution’s normality using the Kolmogorov–Smirnov, skewness, and kurtosis tests [45]. Since the distribution was normal, parametric tests were used. Pearson’s correlation coefficient was calculated to examine the relationship between strength measurement methods. The correlation coefficient is the coefficient that indicates the direction and magnitude of the relationship between independent variables. This coefficient takes a value between (−1) and (+1). Positive values indicate a direct linear relationship; negative values indicate a reverse linear relationship.

## 3. Results

Female athletes had an average age of 15.5 years (±1.57 years), while male athletes had an average age of 14.73 years (±1.14 years). The participants’ average training experience was 4.87 years (±2.26 years), and their average height was 167.42 cm (±9.45 cm).

The analysis of Table 1 suggests that the standing long jump measurement is not significantly correlated (*p* > 0.05) with other strength assessments performed in this study. However, there seems to be a different trend with the Herman trainer measurements. These measurements exhibited statistically significant correlations with the Lafayette measurements, particularly between right and left palding chagi techniques. This correlation has a strong magnitude effect of 0.959. A similar pattern was observed in the dollyeo chagi technique. A significant correlation between the palding chagi and other taekwondo techniques was observed, ranging from 0.405 to 0.642. Additionally, Table 1 indicates a high correlation between hamstring measurements and taekwondo technical strength. The left hamstring strength, for example, strongly correlated with the left palding chagi technique (correlation value of 0.736). This pattern was mirrored in the right hamstring and right palding chagi technique (r = 0.671).

## 4. Discussion

In order to accurately assess sports performance, it’s crucial to use sport-specific measurements that closely replicate training and competition conditions. In taekwondo, as demonstrated in our research, objective performance evaluation requires measuring techniques like the palding, dollyeo chagi, yeop chagi, and dwit chagi. For this study, which compared the effectiveness of different strength measurement methods in taekwondo athletes, we assessed four technical striking strengths using a Herman digital trainer attached to a striking stand. Additionally, we measured quadriceps and hamstring strength with a Lafayette digital hand dynamometer and explosive leg strength through the standing long jump. Prior research supports the importance of these measurements, having examined how various training methods impact strength in taekwondo athletes [46,47,48]. This research offers originality by directly comparing different strength measurement methods. While the standing long jump is commonly used to assess explosive strength in taekwondo athletes [2,49], and while some studies suggest vertical and standing long jumps can be reliable measures of lower-body strength in children and adolescents [50,51], their effectiveness in evaluating sport-specific strength has limitations. Research indicates that the standing long jump may have poor validity in measuring explosive muscle strength, particularly in young males [51]. Our findings support this, with no significant correlation observed between the standing long jump and taekwondo-specific impact force or manual muscle tester values (*p* > 0.05) (Table 1). It may not reflect the specific strength of taekwondo movements, as a poor correlation was found between the results of this test and those of the Herman trainer. This suggests the standing long jump might not adequately reflect the specific strength demands of taekwondo movements, aligning with Hraski’s conclusion that it may be insufficient for predicting strength in this athlete population [51]. In disciplines like taekwondo, sport-specific measurement methods are crucial to objectively assess specialized strength development and effectively monitor athlete progress.

It has been stated that hand dynameters, such as the Lafayette manual muscle testing device, have become useful in athletic field tests because they are a convenient, portable, non-invasive, relatively fast, easy, and inexpensive way to objectively measure applied forces [38]. The Herman trainer is a valuable tool in combat sports like taekwondo, as it assesses physiological effects [52], measures the g-force produced by kicks [53], and helps determine taekwondo athletes’ strike force and technical stroke rates [33]. Our results showed a moderate to high positive correlation between the Herman digital trainer and Lafayette digital hand dynamometer measurements (Table 1). Our findings align with previous research demonstrating interchangeability between isometric (manual muscle tester), isotonic (universal or nautilus), and isokinetic (Cybex II) techniques for measuring knee muscle strength and imbalance in athletes [54]. 

The palding chagi is a front kick to the opponent’s abdomen, a technique known for its potential to dramatically shift the course of a taekwondo competition. Its quick execution allows for surprise points that can change the dynamic of a match. A fast and effective palding chagi requires athletes to have flexible foot, knee, and hip joints [55]. The dollyeo chagi is a variation of the palding, executed as a circular kick aimed at the head. In its final stage, the kicking leg and body align. Trunk rotation speed, along with hip, hamstring, and leg strength, positively influence the technique’s power. With head strikes having a high knockout potential, forceful kicks have a significant impact on competition outcomes [55]. Monitoring these kick strengths, therefore, offers valuable insights for coaches and athletes during training and pre-competition preparation. From this point of view, taking technical kick strength measurements in taekwondo provides useful information to trainers and athletes. In a study conducted in the literature, it was stated that there are differences in the weights of elite taekwondo athletes according to different weight classes, and the effect of the horizontal kick technique on movement speed becomes evident [56]. Athletes can be classified as novice, intermediate, and elite, according to the magnitude of the forces produced in the taekwondo kick of strength and technical strength [18].

A review of the literature reveals conflicting evidence regarding the reliability of digital hand dynamometers—while some studies assert their reliability [57,58,59], others indicate low levels of reliability [60]. For example, in 2015, Mentiplay and colleagues examined the reliability of three different digital hand dynamometers on lower extremity muscle groups and found that digital hand dynamometers were reliable and stated that healthy individuals were especially reliable in isometric measurements of lower extremity muscle groups [57]. Similarly, another study examined the validity and reliability of a belt-mounted digital hand dynamometer in the isometric evaluation of hip muscles and stated that the hand dynamometer is a valid and reliable method for isometric measurements [58]. Another study supporting this stated that digital hand dynamometers are useful and reliable; they examined the validity of the digital hand dynamometer in the evaluation of quadriceps muscle strength by comparing it with the gold standard Biodex isokinetic dynamometer measurements and stated that it was feasible, inexpensive, and portable for quadriceps muscle strength measurement [59]. Arguing instead that the reliability of digital hand dynamometers is low, Toonstra et al.’s study compared three different muscle strength assessment methods on the knee extensor and flexor muscles and found that the reliability of the digital hand dynamometer was low [60]. When the literature is evaluated, it can be seen that dynamic hand dynamometers show high correlations in some muscle groups and low correlations in others, compared to the gold standard isokinetic dynamometers. Additionally, there are studies in the literature showing that dynamic hand dynamometers have high validity and reliability in monitoring muscle strength development and when used with the test–retest method [61]. Kelln et al. stated that they used the test–retest method to measure the validity and reliability of dynamic hand dynamometers in assessing lower extremity muscle strength and that they have the potential to be valid in healthy individuals [39].

The study revealed a positive correlation between manual muscle testing and the Herman trainer, a specialized tool for directly evaluating taekwondo techniques. Such correlation was particularly strong between hamstring strength and palding–dollyeo chagi variations (Table 1). These findings suggest the hamstring muscle plays a greater role in predicting technical strike strength compared to the quadriceps, which is primarily involved in daily activities like walking, running, and stair climbing. This contributes to the scientific field, as technical kick assessments with the Herman trainer are more time-consuming and complex than manual muscle testing. Striking force is widely regarded as a key component for success in combat sports [18,62,63]. Our results shed light on the relationships between three different force measurement methods, potentially offering practical alternatives for assessing specialized strength. In order for taekwondo athletes to score points in the electronic system, their strikes must exceed a specific force threshold determined by their weight category. Therefore, developing sufficient striking strength is crucial for success. While various methods exist to measure strength, it is essential to choose one that accurately assesses the specific demands of the sport. The Herman trainer stands out as particularly effective for taekwondo, as it is directly mounted on the striking stand used in training and directly measures an athlete’s striking force. 

## 5. Conclusions

The results of the standing long jump, which is frequently used as a field test in sportive applications, did not show a correlation with the other strength measurement results used in our study. In branches such as taekwondo, target muscles should be selected for strength assessment and strength measurement methods that can accurately represent the strength of the target area should be used. The positive correlation observed between the Lafayette manual muscle tester and the Herman trainer in our study shows that the results of the tests are consistent with each other. In the literature, there are also studies emphasizing that more accurate results can be obtained in monitoring the development of athletes if strength is measured regularly. In this direction, regular follow-up of athletes for strength measurements is recommended. 

## Figures and Tables

**Table 1:** Pearson correlation coefficient results of the strength measurements.

Variables	1	2	3	4	5	6	7	8	9	10	11	12	13
1. Standing long jump	1												
2. Palding chagi—right	0.050	1											
3. Palding chagi—left	0.077	0.959 **	1										
4. Dollyeo chagi—right	0.132	0.642 **	0.633 **	1									
5. Dollyeo chagi—left	0.132	0.603 **	0.622 **	0.942 **	1								
6. Dwit chagi—right	0.004	0.468 **	0.500 **	0.408 *	0.421 *	1							
7. Dwit chagi—left	0.044	0.446 *	0.534 **	0.393 *	0.418 *	0.797 **	1						
8. Yeop chagi—right	0.101	0.405 *	0.452 *	0.174	0.208	0.384 *	0.455 *	1					
9. Yeop chagi—left	−0.001	0.470 **	0.505 **	0.396 *	0.468 **	0.518 **	0.615 **	0.574 **	1				
10. Quadriceps—right	−0.239	0.333	0.321	0.373 *	0.314	0.284	0.263	0.344	0.250	1			
11. Quadriceps—left	−0.131	0.376 *	0.403 *	0.396 *	0.358 *	0.381 *	0.329	0.335	0.216	0.895 **	1		
12. Hamstring—right	−0.174	0.671 **	0.670 **	0.628 **	0.625 **	0.496 **	0.443 *	0.381 *	0.408 *	0.624 **	0.583 **	1	
13. Hamstring—left	−0.111	0.736 **	0.736 **	0.551 **	0.561 **	0.503 **	0.465 **	0.372 *	0.429 *	0.429 *	0.471 **	0.868 **	1

Note: Data points 2–9 are the results of different impact forces taken with the Herman trainer. Data points 10–13 represent force results obtained with the Lafayette manual tester. Pearson correlation coefficient, * *p* < 0.05, ** *p* < 0.001.

## Data Availability

Data supporting the findings of this study are available from the corresponding author upon reasonable request.
